# Supplementary material for: Predicting the probability of death using proteomics
Source: Commun Biol. 2021 Jun 18;4:758. doi: 10.1038/s42003-021-02289-6 (PMC8213855; doi:10.1038/s42003-021-02289-6)
Supplement: Supplementary file 2 — Supplementary Information [file 42003_2021_2289_MOESM2_ESM.pdf]

Supplementary Tables

Supplementary Table 1: Number of proteins chosen by the Boruta method and the L1 penalized logistic regression models for death within 1,2,...,15, and the overlap between years.

**All participants**						
**Death within years**	**# protein measurements selected by prediction model**	**Overlap with previous year**	**Cumulative overlap with previous years**	**# relevant protein measurements selected by Boruta**	**Overlap with previous year**	**Cumulative overlap with previous years**
**1**	81			209		
**2**	98	39	39	236	178	178
**3**	87	51	23	236	207	163
**4**	94	59	17	235	199	146
**5**	117	72	17	249	210	141
**6**	136	86	17	263	228	139
**7**	156	98	16	390	259	139
**8**	193	134	16	386	362	139
**9**	207	149	14	407	365	137
**10**	199	151	13	407	387	137
**11**	207	158	12	418	392	137
**12**	212	164	11	423	397	137
**13**	219	171	11	439	402	135
**14**	190	151	10	471	426	135
**15**	192	150	10	454	433	135
**Participants older than 60**						
**1**	56			177		
**2**	75	26	26	183	133	133
**3**	75	48	19	185	151	115
**4**	88	55	14	182	158	102
**5**	71	57	13	185	158	95
**6**	93	58	11	180	157	92
**7**	116	75	11	215	167	91
**8**	114	85	11	195	183	87
**9**	106	82	11	206	181	85
**10**	119	85	10	214	185	85
**11**	115	88	8	213	185	83
**12**	116	86	7	212	187	81
**13**	116	87	7	220	193	80
**14**	124	90	7	220	196	78
**15**	115	92	7	229	202	78

Supplementary Table 2: Discrimination and reclassification performance of different models for two-, five- and ten-year prediction

Model	AUC (95% confidence interval)	AUC difference from baseline (p-val)	IDI vs baseline (95% confidence interval)
	**All participants**		
**Death within 2-years**			
Age+sex	0.842(0.819-0.865)	-0.051(4.6E-08)	-0.063(-0.077--0.048)
Baseline	0.894(0.878-0.910)	-	-
Age+sex+GDF15	0.912(0.897-0.927)	0.018(2.9E-02)	0.067(0.040-0.097)
Age+sex+protein	0.932(0.919-0.945)	0.039(9.7E-07)	0.167(0.128-0.206)
**Death within 5-years**			
Age+sex	0.852(0.838-0.865)	-0.033(2.4E-11)	-0.058(-0.069--0.047)
Baseline	0.885(0.872-0.896)	-	-
Age+sex+GDF15	0.893(0.882-0.905)	0.008(8.7E-02)	0.038(0.022-0.055)
Age+sex+protein	0.915(0.903-0.925)	0.030(1.4E-09)	0.115(0.095-0.137)
**Death within 10-years**			
Age+sex	0.883(0.874-0.892)	-0.018(4.7E-12)	-0.043(-0.050--0.036)
Baseline	0.901(0.893-0.909)	-	-
Age+sex+GDF15	0.905(0.897-0.913)	0.004(1.9E-01)	0.015(0.005-0.025)
Age+sex+protein	0.923(0.915-0.931)	0.022(3.1E-13)	0.077(0.065-0.090)
	**Participants older than 60**		
**Death within 2-years**			
Age+sex	0.737(0.702-0.772)	-0.074(5.3E-07)	-0.067(-0.084--0.052)
Baseline	0.811(0.784-0.837)	-	-
Age+sex+GDF15	0.841(0.816-0.868)	0.030(4.4E-02)	0.056(0.031-0.086)
Age+sex+protein	0.874(0.850-0.896)	0.062(5.7E-06)	0.136(0.102-0.172)
**Death within 5-years**			
Age+sex	0.750(0.727-0.772)	-0.050(4.3E-10)	-0.061(-0.072--0.049)
Baseline	0.801(0.781-0.818)	-	-
Age+sex+GDF15	0.820(0.799-0.839)	0.019(3.4E-02)	0.040(0.022-0.058)
Age+sex+protein	0.853(0.835-0.870)	0.053(4.5E-09)	0.113(0.092-0.136)
**Death within 10-years**			
Age+sex	0.774(0.757-0.791)	-0.032(3.8E-10)	-0.049(-0.058--0.041)
Baseline	0.806(0.789-0.822)	-	-
Age+sex+GDF15	0.819(0.803-0.834)	0.013(2.8E-02)	0.022(0.011-0.033)
Age+sex+protein	0.851(0.837-0.864)	0.045(9.0E-13)	0.086(0.072-0.100)

Supplementary Table 3: Discrimination performance with and without the baseline variables included in the models.

	**All participants**			**Participants older than 60**		
	**AUC (95% confidence interval)**		**AUC difference (p-val)**	**AUC (95% confidence interval)**		**AUC difference (p-val)**
**Death within 2-years**	Age+sex+	Baseline+		Age+sex+	Baseline+	
GDF15	0.912 (0.897-0.927)	0.929 (0.916-0.942)	0.017 (4.8E-04)	0.841 (0.816-0.868)	0.868 (0.847-0.893)	0.027 (2.2E-03)
Protein	0.932 (0.919-0.945)	0.938 (0.926-0.951)	0.006 (1.9E-02)	0.874 (0.850-0.896)	0.885 (0.864-0.908)	0.011 (1.4E-02)
**Death within 5-years**	Age+sex+	Baseline+		Age+sex+	Baseline+	
GDF15	0.893 (0.882-0.905)	0.907 (0.896-0.918)	0.014 (5.0E-06)	0.820 (0.799-0.839)	0.839 (0.820-0.857)	0.020 (4.8E-05)
Protein	0.915 (0.903-0.925)	0.921 (0.910-0.930)	0.006 (4.4E-04)	0.853 (0.835-0.870)	0.861 (0.844-0.877)	0.008 (2.3E-03)
**Death within 10-years**	Age+sex+	Baseline+		Age+sex+	Baseline+	
GDF15	0.905 (0.897-0.913)	0.913 (0.906-0.921)	0.008 (4.2E-06)	0.819 (0.803-0.834)	0.830 (0815-0.846)	0.012 (3.5E-04)
Protein	0.923 (0.915-0.931)	0.926 (0.918-0.933)	0.003 (8.3E-03)	0.851 (0.837-0.864)	0.856 (0842-0.869)	0.005 (9.7E-03)

Supplementary Table 4: Top ten single protein associations with all-cause mortality for different time to death.

Proteins	Gene Name	Beta	P-value
**Death within 2 years**			
Growth/differentiation factor 15	GDF15	1.35	2.75E-137
Thrombospondin-2	THBS2	0.76	8.33E-99
Alpha-1-antichymotrypsin complex	SERPINA3	0.79	5.60E-91
Retinoblastoma-like protein 2	RBL2	0.84	5.97E-87
WAP four-disulfide core domain protein 2	WFDC2	0.92	4.62E-85
Spondin-2	SPON2	0.92	1.41E-82
Serum amyloid A-1 protein	SAA1	0.67	8.38E-82
Serum amyloid A-2 protein	SAA2	0.54	2.26E-80
C5a anaphylatoxin	C5	0.93	1.78E-78
Anthrax toxin receptor 2	ANTXR2	-0.67	8.11E-76
**Death within 5 years**			
Growth/differentiation factor 15	GDF15	1.20	1.91E-167
WAP four-disulfide core domain protein 2	WFDC2	0.82	2.05E-111
Thrombospondin-2	THBS2	0.62	9.07E-100
Anthrax toxin receptor 2	ANTXR2	-0.56	4.62E-88
Retinoblastoma-like protein 2	RBL2	0.65	9.23E-88
Alpha-1-antichymotrypsin complex	SERPINA3	0.61	1.51E-86
Tumor necrosis factor receptor superfamily member 1A	TNFRSF1A	0.64	9.82E-85
Angiopoietin-2	ANGPT2	0.64	1.11E-82
Macrophage metalloelastase	MMP12	0.66	3.94E-81
Spondin-2	SPON2	0.67	1.40E-80
**Death within 10 years**			
Growth/differentiation factor 15	GDF15	1.04	1.45E-155
WAP four-disulfide core domain protein 2	WFDC2	0.76	1.01E-116
Macrophage metalloelastase	MMP12	0.62	1.22E-86
Thrombospondin-2	THBS2	0.50	6.10E-81
Angiopoietin-2	ANGPT2	0.55	9.47E-79
Retinoblastoma-like protein 2	RBL2	0.54	1.22E-78
Tumor necrosis factor receptor superfamily member 1A	TNFRSF1A	0.56	4.49E-77
Alpha-1-antichymotrypsin complex	SERPINA3	0.50	3.89E-75
Anthrax toxin receptor 2	ANTXR2	-0.45	2.93E-73
Transgelin	TAGLN	0.65	8.46E-67
**Death within 15 years**			
Growth/differentiation factor 15	GDF15	1.03	3.38E-154
WAP four-disulfide core domain protein 2	WFDC2	0.74	3.76E-114
Macrophage metalloelastase	MMP12	0.62	7.05E-85
Angiopoietin-2	ANGPT2	0.54	6.20E-79
Thrombospondin-2	THBS2	0.46	6.69E-69
Retinoblastoma-like protein 2	RBL2	0.48	3.58E-67
Anthrax toxin receptor 2	ANTXR2	-0.42	3.42E-65
Tumor necrosis factor receptor superfamily member 1A	TNFRSF1A	0.50	3.29E-63
Sushi, von Willebrand factor type A, EGF and pentraxin domain-containing protein 1	SVEP1	0.51	1.64E-60
C5a anaphylatoxin	C5	0.44	2.43E-58

Supplementary Table 5: Top single protein associations with different causes of death within five years.

Protein	Gene Name	Beta	P-value
**Neoplasms**			
Growth/differentiation factor 15	GDF15	1.42	3.83E-140
Thrombospondin-2	THBS2	0.77	2.02E-90
Retinoblastoma-like protein 2	RBL2	0.85	2.29E-88
WAP four-disulfide core domain protein 2	WFDC2	0.88	6.96E-83
Alpha-1-antichymotrypsin complex	SERPINA3	0.75	5.92E-77
**Nervous system**			
Hematopoietic prostaglandin D synthase	HPGDS	-0.66	9.24E-08
Anthrax toxin receptor 2	ANTXR2	-0.60	1.41E-07
Disintegrin and metalloproteinase domain-containing protein 22	ADAM22	-0.99	5.86E-07
Growth hormone receptor	GHR	-0.72	9.83E-07
Insulin-like growth factor-binding protein 2	IGFBP2	0.86	2.55E-06
**Circulatory system**			
Growth/differentiation factor 15	GDF15	1.01	1.03E-42
WAP four-disulfide core domain protein 2	WFDC2	0.85	8.93E-42
N-terminal pro-BNP	NPPB	0.66	2.73E-34
Transmembrane emp24 domain-containing protein 10	TMED10	0.53	5.62E-32
Ribonuclease pancreatic	RNASE1	0.57	6.63E-32
**Respiratory system**			
WAP four-disulfide core domain protein 2	WFDC2	1.12	2.96E-21
Growth/differentiation factor 15	GDF15	1.12	1.32E-16
Troponin T. cardiac muscle	TNNT2	0.62	1.02E-14
Cartilage intermediate layer protein 2	CILP2	-0.82	4.48E-13
Hematopoietic prostaglandin D synthase	HPGDS	-0.72	7.09E-13
**Other**			
Growth/differentiation factor 15	GDF15	1.13	3.05E-22
Tyrosine-protein kinase transmembrane receptor ROR2	ROR2	0.53	6.28E-19
WAP four-disulfide core domain protein 2	WFDC2	0.85	1.35E-18
Beta-2-microglobulin	B2M	0.50	1.77E-18
Tumor necrosis factor receptor superfamily member 1A	TNFRSF1A	0.66	1.22E-17

Supplementary Table 6: Order of proteins selected into an all-cause mortality prediction model on top of age and sex.

**Death within 5 years**	**Gene Name**	**Forward selection order**	**L1 bootstrap order**	**Included in 1000 bootstraps**
Growth/differentiation factor 15	GDF15	1	6	997
Anthrax toxin receptor 2	ANTXR2	2	81	801
Insulin-like growth factor-binding protein 2	IGFBP2	3	161	656
Serum amyloid A-1 protein	SAA1	4	12	990
Insulin-like growth factor-binding protein 6	IGFBP6	5	7	997
Spondin-1	SPON1	6	182	624
Tetranectin	CLEC3B	7	1	1000
dCTP pyrophosphatase 1	DCTPP1	8	2	999
Transgelin	TAGLN	9	31	945
Tumor necrosis factor receptor superfamily member EDAR	EDAR	10	49	902
Alpha-1-antichymotrypsin complex	SERPINA3	11	21	976
Erythropoietin	EPO	12	5	998
Hematopoietic prostaglandin D synthase	HPGDS	13	8	995
Macrophage metalloelastase	MMP12	14	4	999
Complement component C7	C7	15	54	874
L-Selectin	SELL	16	25	969
Netrin receptor UNC5B	UNC5B	17	16	985
Brorin	VWC2	18	34	941
WAP four-disulfide core domain protein 2	WFDC2	19	24	970
Serum amyloid A-2 protein	SAA2	20	167	650
**Death within 10 years**				
Growth/differentiation factor 15	GDF15	1	25	980
Anthrax toxin receptor 2	ANTXR2	2	79	865
Insulin-like growth factor-binding protein 2	IGFBP2	3	302	538
Alpha-1-antichymotrypsin complex	SERPINA3	4	19	988
Epidermal growth factor receptor	EGFR	5	14	996
Sushi, von Willebrand factor type A, EGF and pentraxin domain-containing protein 1	SVEP1	6	350	449
Tetranectin	CLEC3B	7	84	857
Spondin-1	SPON1	8	229	622
Serine protease inhibitor Kazal-type 5	SPINK5	9	9	998
WAP four-disulfide core domain protein 2	WFDC2	10	5	999
Immunoglobulin superfamily member 3	IGSF3	11	11	997
Fc receptor-like protein 1	FCRL1	12	22	985
Pleiotrophin	PTN	13	34	962
RGM domain family member B	RGMB	14	17	991
Ephrin type-B receptor 2	EPHB2	15	8	999
Transgelin	TAGLN	16	66	888
Triggering receptor expressed on myeloid cells 1	TREM1	17	77	867
Elafin	PI3	18	3	1000
WNT1-inducible-signaling pathway protein 2	WISP2	19	18	990
N-terminal pro-BNP	NPPB	20	24	983

Supplementary Table 7: Associations of five-year mortality risk predicted by the protein model and corrected for age and sex with binary risk-factors in the dHS and VSP2 datasets.

	**All participants**				**Participants older than 60**			
**Phenotype**	**Number of controls**	**Number of cases**	**Increase in mean risk in sd.**	**P-value**	**Number of controls**	**Number of cases**	**Increase in mean risk in sd.**	**P-value**
**Diabetes**	14917	695	0.89	2.7E-118	5819	476	0.82	1.2E-67
**MI**	14969	643	0.62	6.5E-54	5002	1293	0.36	1.7E-30
**CAD**	14098	1514	0.40	4.7E-50	5779	516	0.48	4.9E-26
**Cancer**	14401	1211	0.33	2.7E-28	5376	919	0.28	9.7E-15
**Smoker**	14147	1465	0.33	5.1E-34	5750	545	0.40	8.5E-19
**Clonal haematopoiesis**	7518	587	0.09	2.8E-02	3022	474	0.04	3.3E-01

Supplementary Figures

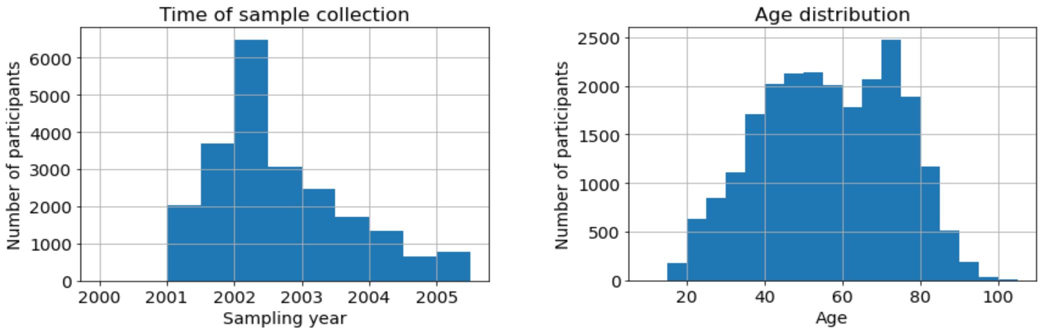

Supplementary Figure 1: Year of plasma sample collection and age distribution in the ICP + VSP1 dataset.

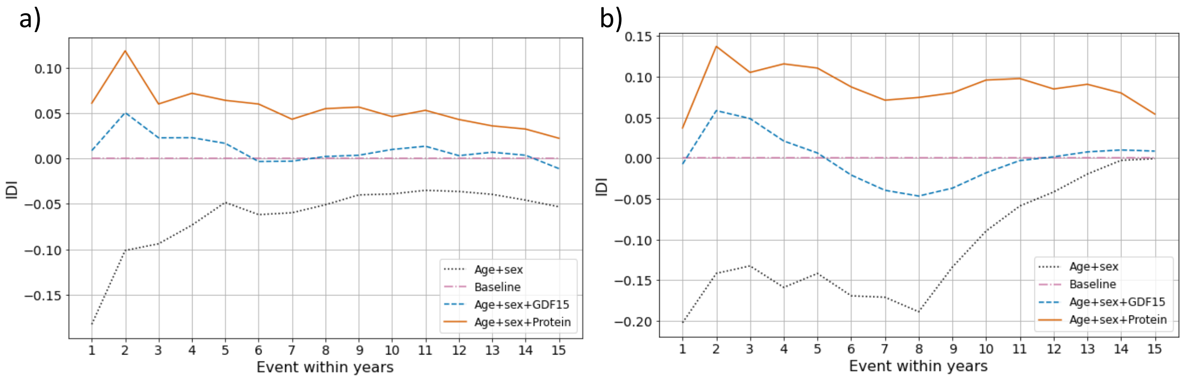

Supplementary Figure 2: IDIs over the baseline at different time points. a) All participants b) Participants older than 60.

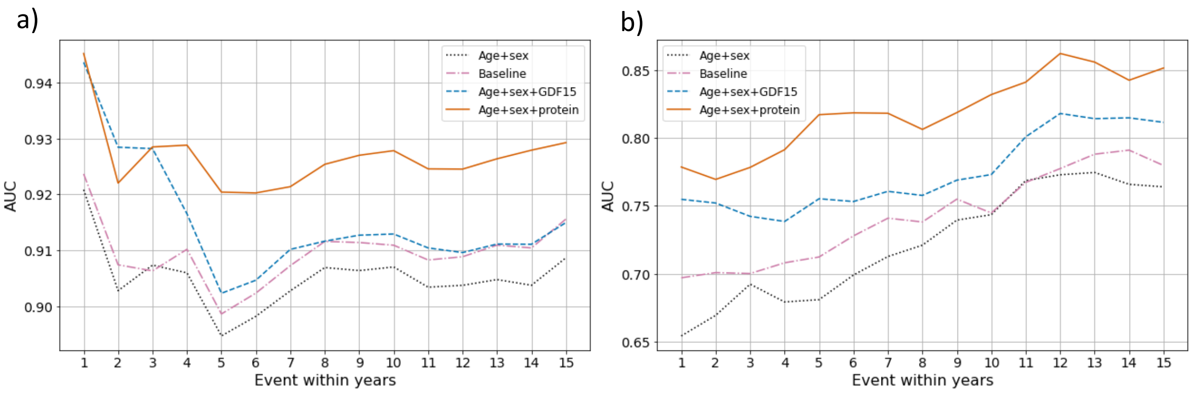

Supplementary Figure 3: AUC at different time points in subsets of the test data. a) All participants not diagnosed with major diseases (Cancer, history of MI, history of stroke, or CAD) at time of plasma collection, N=4,770, b) participants older than 80, N=564.

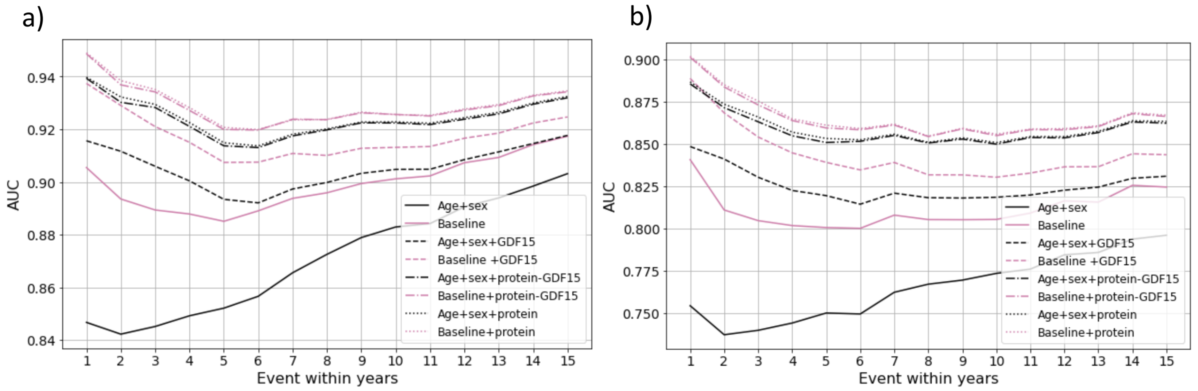

Supplementary Figure 4: AUC at different time points for models with and without baseline features and with and without GDF15. a) All participants, b) participants older than 60.

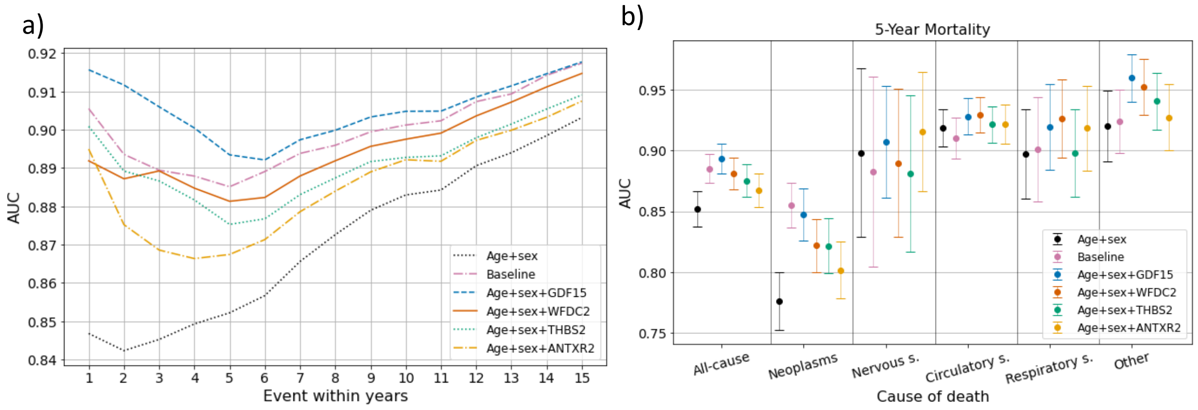

Supplementary Figure 5: AUC for models using single proteins. a) Prediction for different time points, b) five-year prediction for different causes of death, the error bars represent 95% confidence intervals.

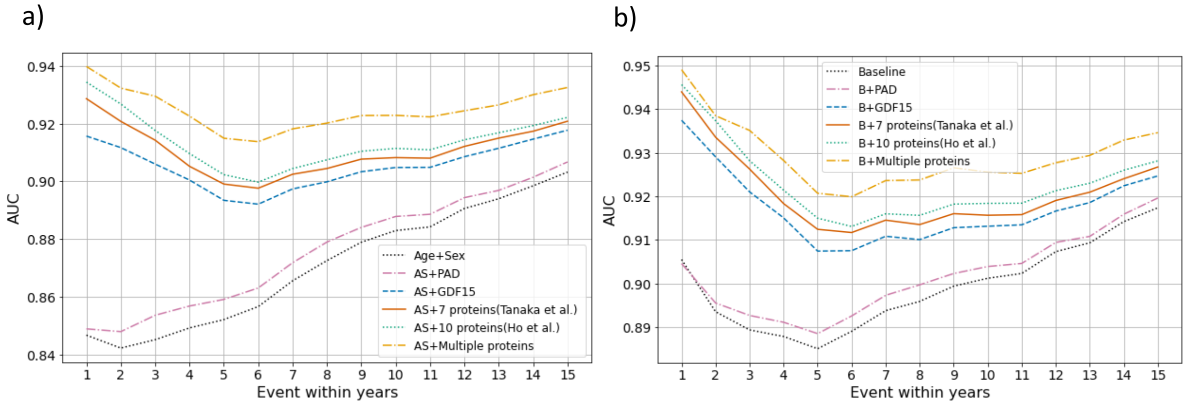

Supplementary Figure 6: AUC for protein models from other studies, at different time points, compared to our models. The figures compare our model which uses multiple proteins and different number of proteins for each time point, to models using combinations of proteins or protein derived PAD identified as good predictors in previous studies. a) Every model includes age and sex, b) every model includes the baseline.

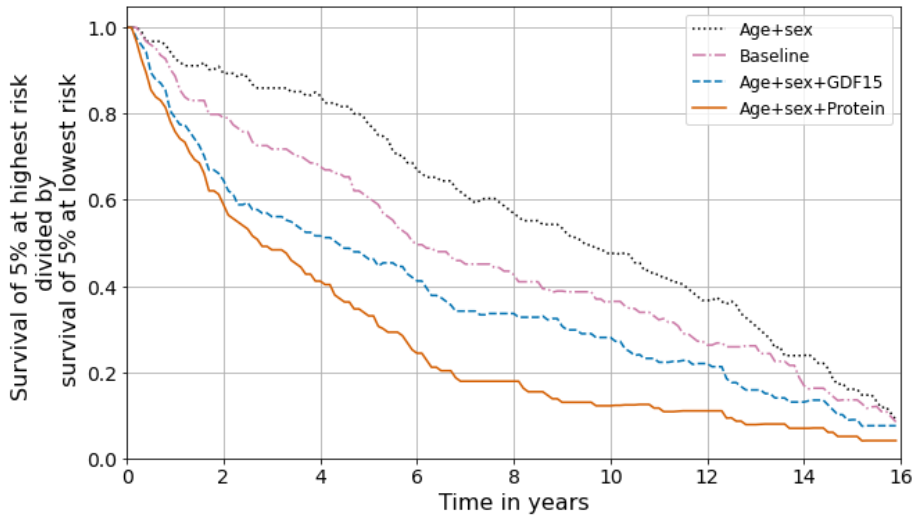

Supplementary Figure 7: Survival of 60-80 years old participants. The Kaplan-Meier survival for the 5% at highest ten-year risk divided by the 5% at the lowest ten-year risk.

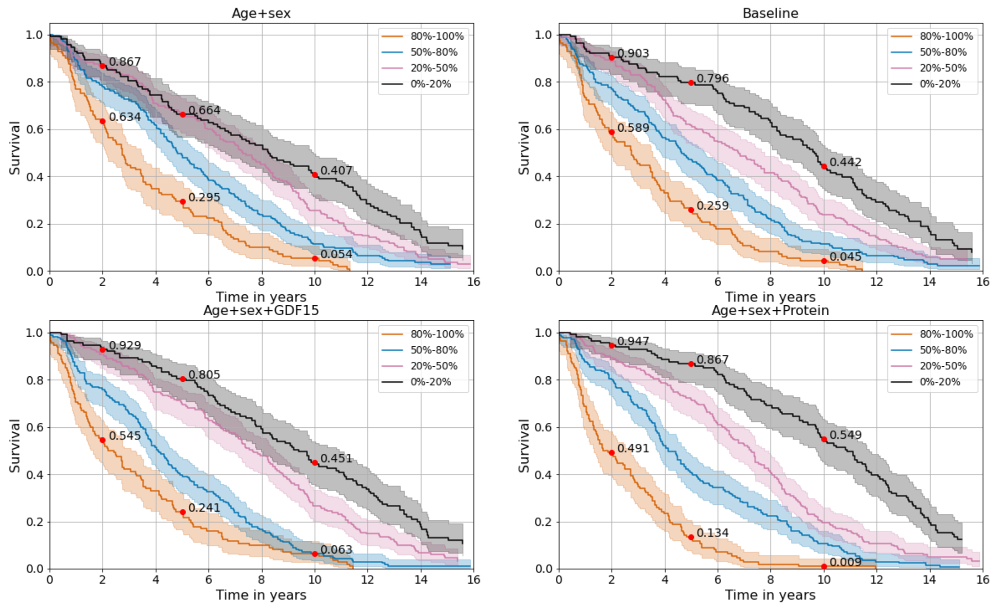

Supplementary Figure 8: Survial curves for participants 80 years old or older, N= 564, split by ten-year predicted risk quantiles. The coloured areas represent 95% confidence intervals.

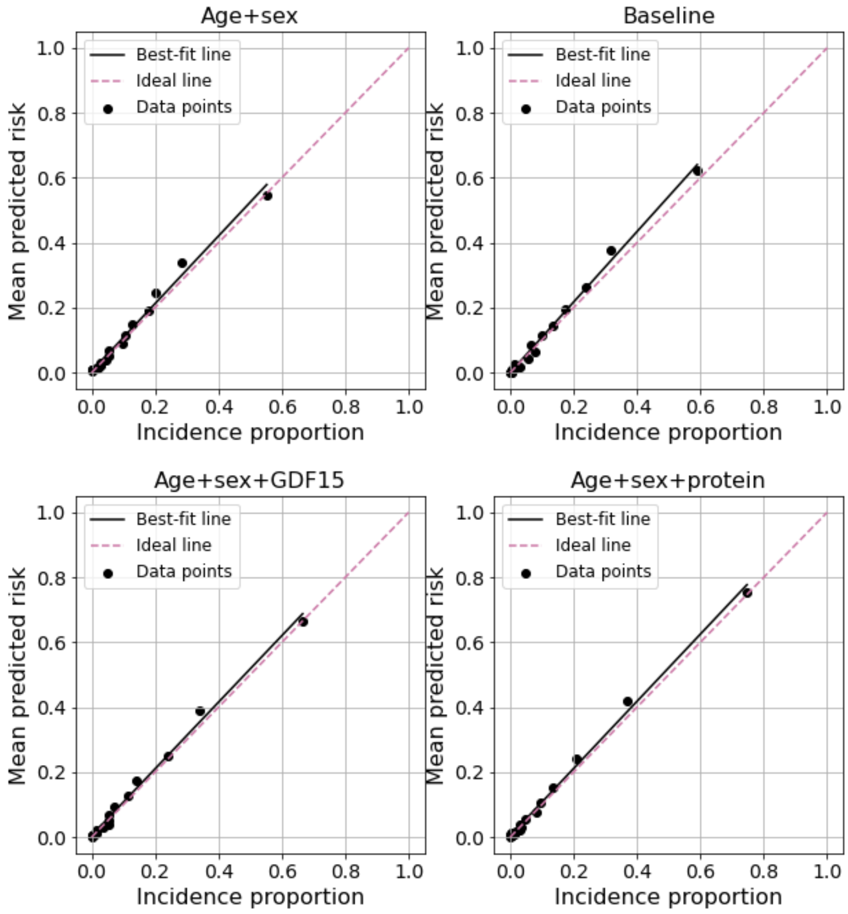

Supplementary Figure 9: Calibration of five-year mortality risk prediction models.

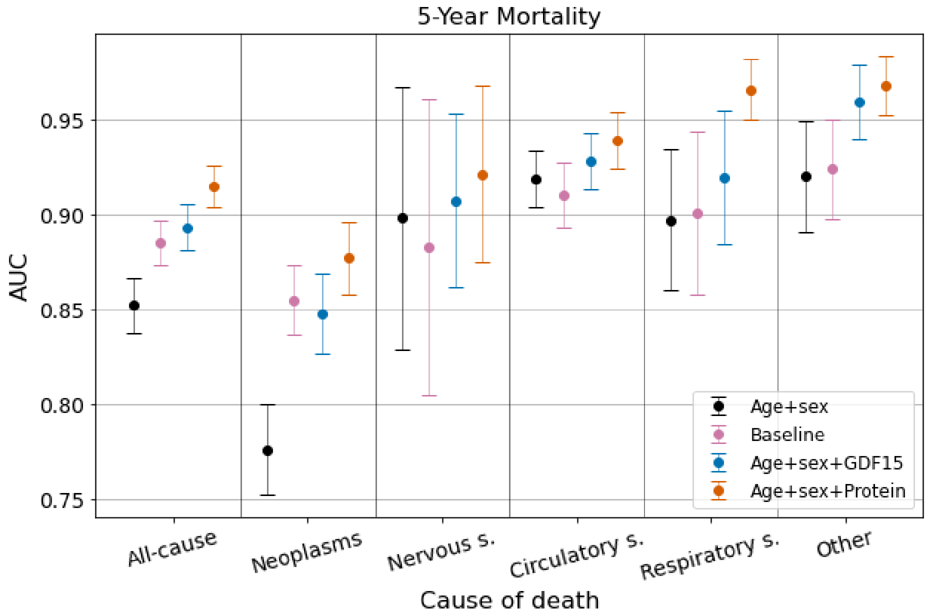

Supplementary Figure 10: AUCs for different causes of death within five years. The AUC is calculated for each cause of death vs. alive, excluding all other causes. The error bars represent 95% confidence intervals.

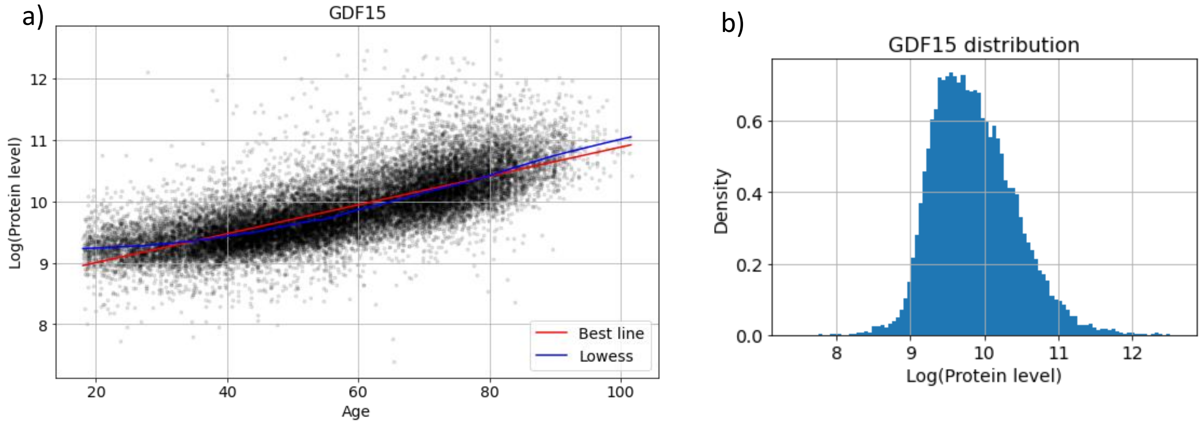

Supplementary Figure 11: GDF15 in the ICP+VSP1 dataset. a) Levels of GDF15 vs. age. The figure also shows the best straight line and a line fit with locally weighted scatterplot smoothing. b) The distribution of GDF levels.

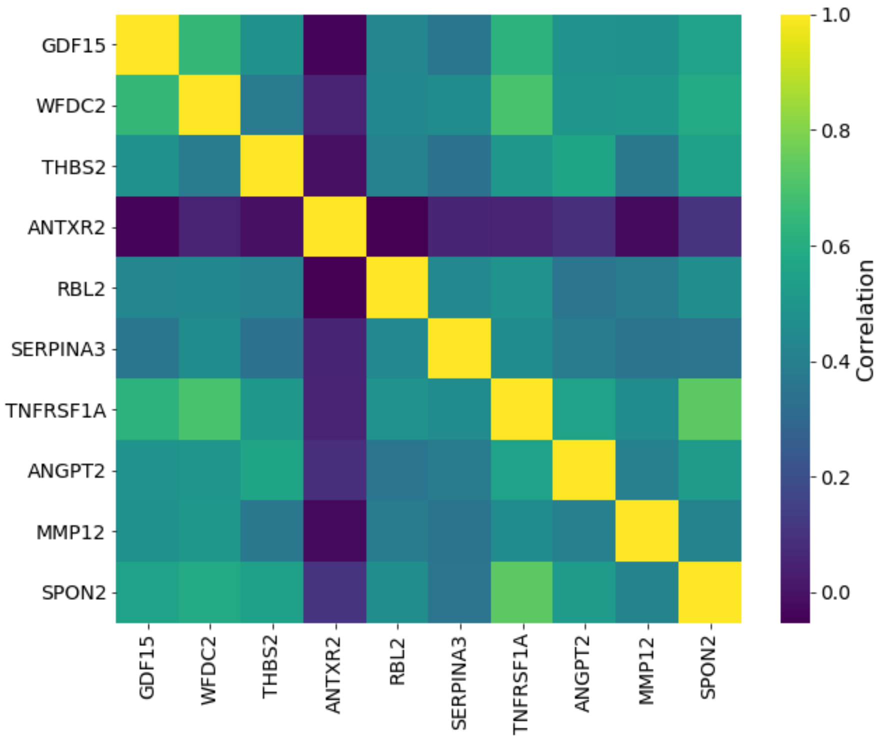

Supplementary Figure 12: Correlations between plasma protein levels for the top ten proteins associated with five-year mortality.

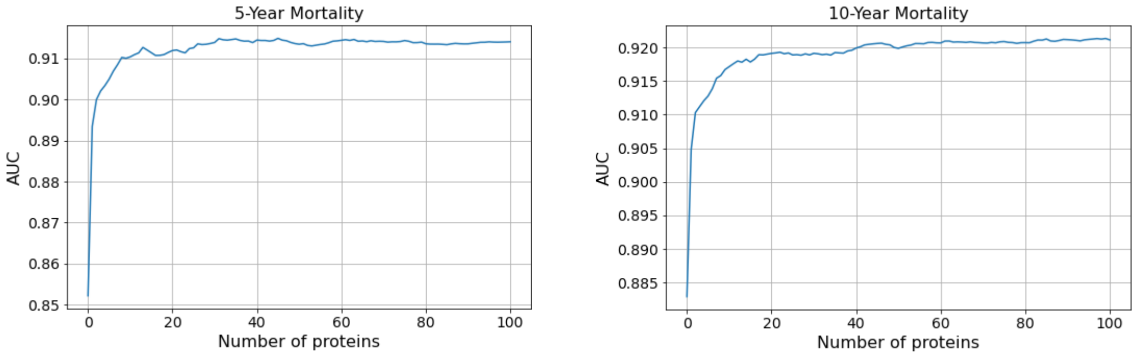

Supplementary Figure 13: AUC for models using age and sex and 0-100 proteins as selected by stepwise forward selection.

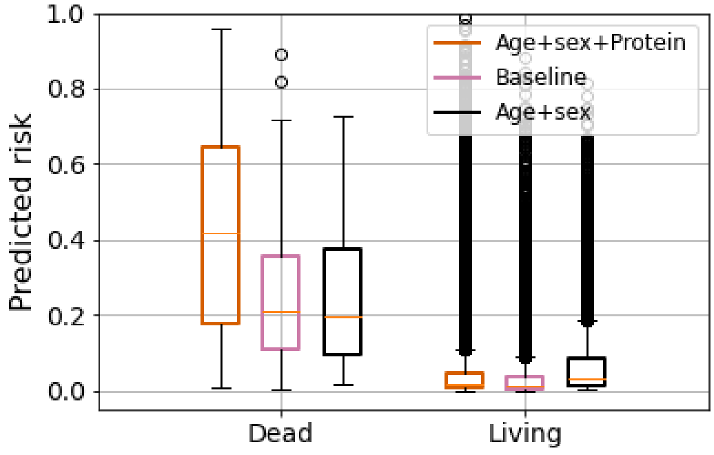

Supplementary Figure 14: Predicted five-year risk for participants in the dHS and VSP2 datasets. The yellow centre line represent the median, the box limits represent the upper and lower quartiles, the whiskers represent the 1.5x interquartile range, and the circles are data points outside the whisker range.

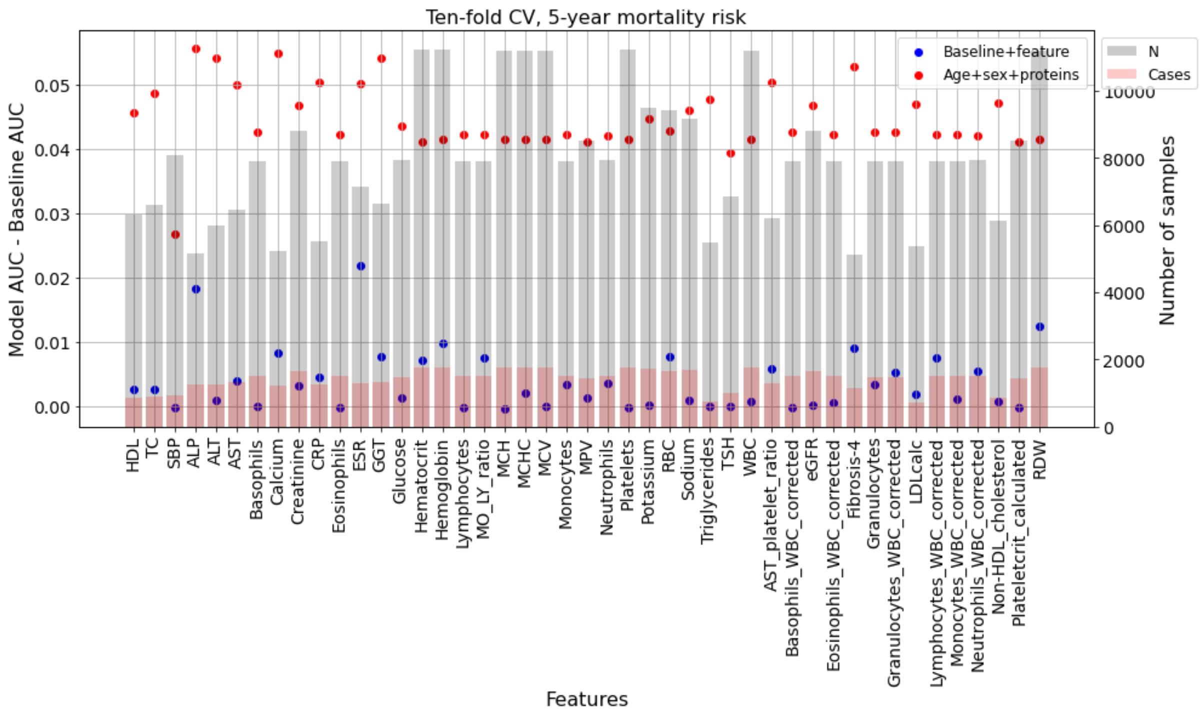

Supplementary Figure 15: Extra features in the baseline model. The blue dots show the increase of mean AUC from including the features on the x-axis in the baseline model. The bars show for how many participants the features are available. Since the AUC is heavily influenced by the dataset, the results for a protein model trained on the same data is also shown (red dots).

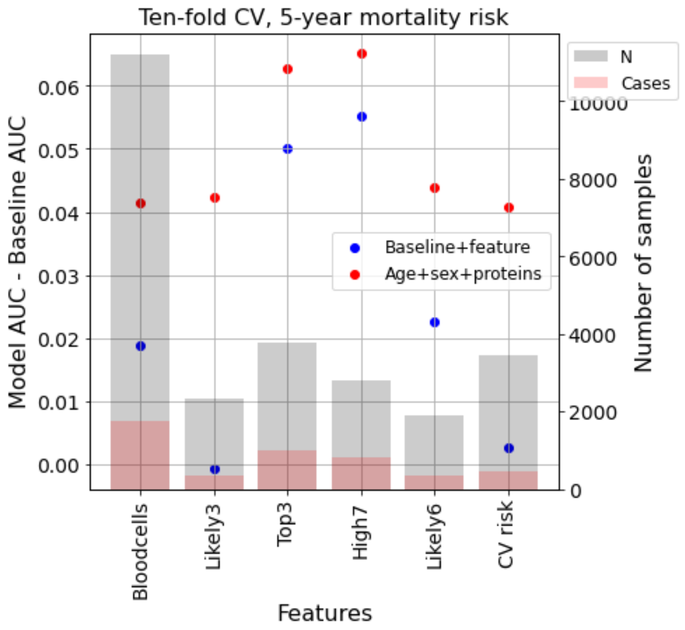

*Supplementary Figure 16: Sets of extra features for the baseline model. The blue dots show the increase of mean AUC from including the set of features in the baseline model. The bars show for how many participants all the features in the set are available. Since the AUC is heavily influenced by the dataset, the results for a protein model trained on the same data is also shown (red dots). **Bloodcells**: Hematocrit, Hemoglobin, MCH, MCHC, Platelets, WBC, and RDW. **Likely3:** SBP, Glucose, and Triglycerides. **Top3:** ALP, ESR, and RDW. **High7:** ALP, ESR, RDW, Fibrosis-4, Hematocrit, Hemoglobin, and RBC. **Likely6:** SBP, Glucose, Triglycerides, HDL, TC, and Creatinine. **CV risk:** SBP, HDL, TC.*

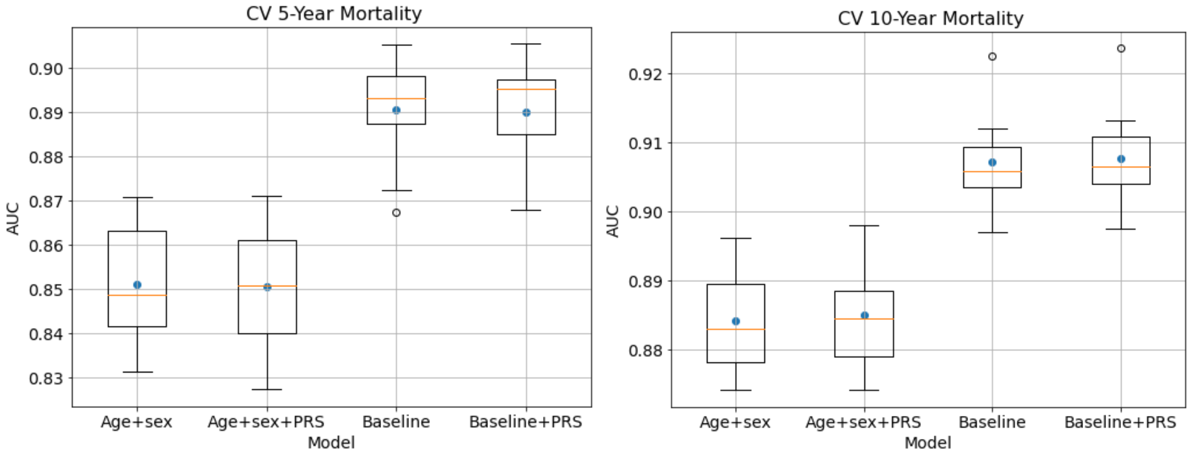

Supplementary Figure 17: Ten-fold CV AUC for models with and without PRS. The yellow centre line represents the median, the box limits represent the upper and lower quartiles, the whiskers represent the 1.5x interquartile range, the circles are data points outside the whisker range, and the blue dots represent the mean values.

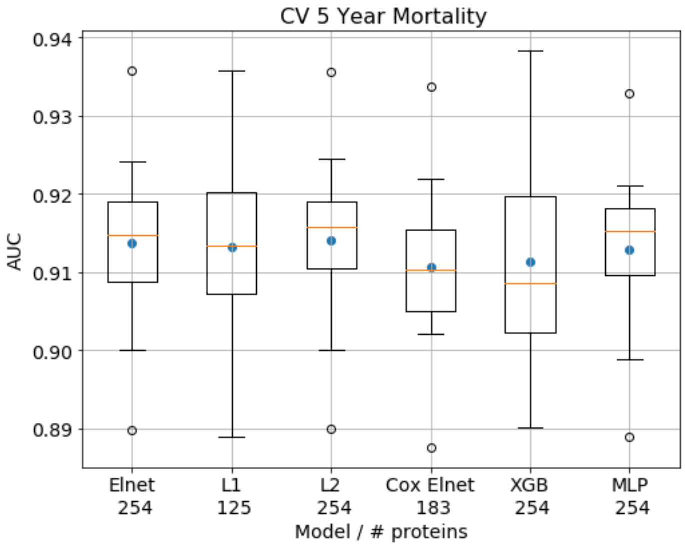

Supplementary Figure 18: Ten-fold CV of five-year mortality risk AUC for different protein models. The yellow centre line represents the median, the box limits represent the upper and lower quartiles, the whiskers represent the 1.5x interquartile range, the circles are data points outside the whisker range, and the blue dots represent the mean values.
