# Supplementary material for: Predicting the probability of death using proteomics
Source: Commun Biol. 2021 Jun 18;4:758. doi: 10.1038/s42003-021-02289-6 (PMC8213855; doi:10.1038/s42003-021-02289-6)
Supplement: Supplementary file 3 — Description of Supplementary Files [file 42003_2021_2289_MOESM3_ESM.pdf]

**Description of Additional Supplementary Files**

**File name:** Supplementary Data 1

**Description:** Coefficient of the five-year all-cause mortality prediction model.

**File name:** Supplementary Data 2

**Description:** Single protein associations between protein levels and five-year mortality risk.

**File name:** Supplementary Data 3

**Description:** Single protein associations between protein levels and five-year mortality risk in participants older than 60.

**File name:** Supplementary Data 4

**Description:** Single protein associations between protein levels and mortality risk using the cox proportional hazards model.

**File name:** Supplementary Data 5

**Description:** Top results from protein pathway analysis.

**File name:** Supplementary Data 6

**Description:** Available measurements of baseline variables in all datasets.

**File name:** Supplementary Data 7

**Description:** Source data underlying all figures in the manuscript.
