# Supplementary material for: Predicting the probability of death using proteomics
Source: Commun Biol. 2021 Jun 18;4:758. doi: 10.1038/s42003-021-02289-6 (PMC8213855; doi:10.1038/s42003-021-02289-6)
Supplement: Supplementary file 11 — Reporting Summary [file 42003_2021_2289_MOESM11_ESM.pdf]

nature research

Corresponding author(s): Magnús Örn Úlfarsson
Kári Stefánsson

Last updated by author(s): 29/04/21

Reporting Summary

Nature Research wishes to improve the reproducibility of the work that we publish. This form provides structure for consistency and transparency in reporting. For further information on Nature Research policies, see our Editorial Policies and the Editorial Policy Checklist.

Statistics

For all statistical analyses, confirm that the following items are present in the figure legend, table legend, main text, or Methods section.

n/a	Confirmed	
☐	☒	The exact sample size (*n*) for each experimental group/condition, given as a discrete number and unit of measurement
☐	☒	A statement on whether measurements were taken from distinct samples or whether the same sample was measured repeatedly
☐	☒	The statistical test(s) used AND whether they are one- or two-sided<br>*Only common tests should be described solely by name; describe more complex techniques in the Methods section.*
☐	☒	A description of all covariates tested
☐	☒	A description of any assumptions or corrections, such as tests of normality and adjustment for multiple comparisons
☐	☒	A full description of the statistical parameters including central tendency (e.g. means) or other basic estimates (e.g. regression coefficient) AND variation (e.g. standard deviation) or associated estimates of uncertainty (e.g. confidence intervals)
☐	☒	For null hypothesis testing, the test statistic (e.g. *F*, *t*, *r*) with confidence intervals, effect sizes, degrees of freedom and *P* value noted<br>*Give P values as exact values whenever suitable.*
☒	☐	For Bayesian analysis, information on the choice of priors and Markov chain Monte Carlo settings
☒	☐	For hierarchical and complex designs, identification of the appropriate level for tests and full reporting of outcomes
☐	☒	Estimates of effect sizes (e.g. Cohen's *d*, Pearson's *r*), indicating how they were calculated

Our web collection on statistics for biologists contains articles on many of the points above.

Software and code

Policy information about availability of computer code

Data collection	Not applicable
Data analysis	All data preparations, plotting, and model training was done in Python version 3.6.3. The logistic regression models were implemented using the machine learning library scikit-learn. Some metrics were calculated in R version 3.6.0 with the package pROC.

For manuscripts utilizing custom algorithms or software that are central to the research but not yet described in published literature, software must be made available to editors and reviewers. We strongly encourage code deposition in a community repository (e.g. GitHub). See the Nature Research guidelines for submitting code & software for further information.

Data

Policy information about availability of data

All manuscripts must include a data availability statement. This statement should provide the following information, where applicable:
- Accession codes, unique identifiers, or web links for publicly available datasets
- A list of figures that have associated raw data
- A description of any restrictions on data availability

We declare that the data supporting the findings of this study are available within the article, its supplementary information, and upon reasonable request. Individual-level data used in this publication are not publicly available because Icelandic law and the regulations of the Icelandic Data Protection Authority prohibit the release of individual-level and personally-identifying data.

Field-specific reporting

Please select the one below that is the best fit for your research. If you are not sure, read the appropriate sections before making your selection.

[x] Life sciences   [ ] Behavioural & social sciences   [ ] Ecological, evolutionary & environmental sciences

For a reference copy of the document with all sections, see nature.com/documents/nr-reporting-summary-flat.pdf

Life sciences study design

All studies must disclose on these points even when the disclosure is negative.

Sample size	The main dataset consisted of 22,913 individuals.<br>Additionally we had 8,814 + 6,798 individuals with little follow up used for validation experiments .
Data exclusions	Pregnant women, individuals who died from external causes, and participants younger than 18 were excluded from the study.
Replication	We had no independent dataset to fully replicate our results but we had a dataset of 8,814 individuals sampled at a different time where we could verify that our predictions correlated with frailty phenotypes.
Randomization	We randomly split our main dataset into training(70%) and testing(30%).
Blinding	Protein levels were measured without information about the samples.

Reporting for specific materials, systems and methods

We require information from authors about some types of materials, experimental systems and methods used in many studies. Here, indicate whether each material, system or method listed is relevant to your study. If you are not sure if a list item applies to your research, read the appropriate section before selecting a response.

Materials & experimental systems

n/a	Involved in the study
[x]	[ ] Antibodies
[x]	[ ] Eukaryotic cell lines
[x]	[ ] Palaeontology and archaeology
[x]	[ ] Animals and other organisms
[ ]	[x] Human research participants
[x]	[ ] Clinical data
[x]	[ ] Dual use research of concern

Methods

n/a	Involved in the study
[x]	[ ] ChIP-seq
[x]	[ ] Flow cytometry
[x]	[ ] MRI-based neuroimaging

Human research participants

Policy information about studies involving human research participants

Population characteristics	The study population characteristics are available in Table 1 of the manuscript.
Recruitment	A large proportion of the participants were recruited for cancer research, therefore, the prevalence of cancer is tha dataset is higher than in the population.
Ethics oversight	All participants who donated samples gave informed consent and the National Bioethics Committee of Iceland approved the study which was conducted in agreement with conditions issued by the Data Protection Authority of Iceland (VSN_14-015, VSN_15-130, and VSN_15-214). Personal identities of the participant's data and biological samples were encrypted by a third-party system (Identity Protection System), approved and monitored by the Data Protection Authority.

Note that full information on the approval of the study protocol must also be provided in the manuscript.
